# Wound Induced Hair Neogenesis – A Novel Paradigm for Studying Regeneration and Aging

**DOI:** 10.3389/fcell.2020.582346

**Published:** 2020-10-15

**Authors:** Myan Bhoopalam, Luis A. Garza, Sashank K. Reddy

**Affiliations:** ^1^Department of Plastic and Reconstructive Surgery, Johns Hopkins University School of Medicine, Baltimore, MD, United States; ^2^Department of Dermatology, Johns Hopkins University School of Medicine, Baltimore, MD, United States

**Keywords:** wound-induced hair follicle neogenesis, aging, hair follicle stem cells, hair follicle neogenesis, Wnt, STAT3, regeneration

## Abstract

Hair follicles are the signature dermal appendage of mammals. They can be thought of as mini-organs with defined polarity, distinct constituent cell types, dedicated neurovascular supply, and specific stem cell compartments. Strikingly, some mammals show a capacity for adult hair follicle regeneration in a phenomenon known as wound-induced hair neogenesis (WIHN). In WIHN functional hair follicles reemerge during healing of large cutaneous wounds, and they can be counted to provide an index of regeneration. While age-related decline in hair follicle number and cycling are widely appreciated in normal physiology, it is less clear whether hair follicle regeneration also diminishes with age. WIHN provides an extraordinary quantitative system to address questions of mammalian regeneration and aging. Here we review cellular and molecular underpinnings of WIHN, explore known age-related changes to these elements, and present unanswered questions for future exploration.

## Introduction

Regeneration in mammalian systems is limited when compared to those of other phyla. In making this comparison, we distinguish between renewal and regeneration. Many mammalian tissues are capable of renewal in response to physiological need or limited damage. Epithelia of skin, lung, and gastrointestinal tract for example, turn over regularly to replace cells at the surface. However, regeneration of complex tissue or organ architectures in mammals is rare. Urodele salamanders can famously regenerate missing limbs, zebrafish can replace cardiac muscle, and planarians can reconstitute most of the body plan from small vestiges. Notwithstanding exceptions like significant skin regeneration in African spiny mouse and limited liver regeneration, such complex organ regeneration is not evident in mammals (Seifert et al., 2012).

A recently characterized process of hair follicle regeneration – wound induced hair neogenesis (WIHN) – offers an additional window into mammalian regeneration. WIHN is characterized by the emergence of hair follicles in the center of large full-thickness cutaneous wounds in mice, rats, and rabbits (Wier and Garza, 2020). While cutaneous wounds in many mammals including humans heal by fibrotic contracture and scarring, WIHN can produce healthy bilaminar skin, hair follicles, and subdermal supports including adipocytes (Plikus et al., 2017). The regeneration of hair follicles during WIHN is particularly striking, as these key dermal appendages were thought to arise only in embryonic development. In their seminal cellular and molecular characterization of WIHN, Ito et al. (2007) showed that these hair follicles truly arise *de novo* in adults in a process that recapitulates key steps in embryonic hair follicle development.

Hair follicles can be thought of as organs in miniature with their defined polarity, distinct cell types, dedicated neurovascular supply, and stem cell compartments that support continued renewal. True regeneration of such complex structures is rare in mammals, establishing the WIHN system as an important tool to uncover principles of mammalian regeneration. A further advantage of studying WIHN is the ability to easily count novel hair follicles. This provides a quantitative index of regeneration by which to test perturbations that may inhibit or stimulate the process. The WIHN system may be particularly good at addressing an enduring mystery in mammalian regeneration – the extent to which regenerative capacity declines with age. Below we review critical cellular and molecular processes underlying WIHN and explore age-dependent changes in those processes.

## Overview of WIHN and Aging

The healing of wounds with hair follicle regeneration in rodents and rabbits was first uncovered in the 1950s (Lacassagne and Latarjet, 1946; Breedis, 1954; Billingham and Russell, 1956; Chuong, 2007). The process was rediscovered and characterized in cellular and molecular terms in 2007 in a publication by Ito et al. that termed it wound induced hair neogenesis (WIHN). This group discovered that hair follicles emerge 2–3 weeks after full-thickness cutaneous wounds are created on common laboratory mouse strains (*Mus musculus*). Intriguingly, only large wounds (>1 cm^2^) exhibited WIHN, whereas smaller wounds contracted in a fashion similar to larger ones but with the absence of hair follicle generation. Furthermore, hair follicles were only found in the center of these large wounds, not in the periphery. Morphologically, *de novo* hair follicles exhibited nearly all features of neighboring follicles in unwounded skin. The WIHN follicles were similarly polarized, associated with blood vessels, nerves, and sebaceous glands, and were embedded in dermis with subdermal adipocytes. The bulge region of the *de novo* follicles was populated by stem cells capable of supporting subsequent cycling. Differences from follicles generated during embryonic development were few, including an absence of melanocytes and erector pili muscles (Ito et al., 2007). Interestingly, African spiny mice (*Acomys cahirinus*) show even more robust WIHN and can regenerate erector pili muscles as well.

In Ito’s original description of WIHN no significant differences were found in *de novo* hair forming capacity of young (3 week or 7–8 week) or middle-aged (10 month) mice when initial wound size was adjusted to achieve a similarly sized final healed area. It was noted in these original studies that efficient WIHN did not occur in wounds smaller than 1 cm^2^. By contrast, in examining smaller initial wounds much earlier in life, Rognoni et al. (2016) uncovered a major difference in the ability of neonatal mice to undergo WIHN compared to even slightly older counterparts. Specifically, P2 mice exhibited significant hair follicle neogenesis in response to small (2 mm) wounds, whereas this decreased in age-dependent manner plateauing at P21 (Rognoni et al., 2016). Thus, in the immediate post-natal period, WIHN appears more robust than it does in even young animals. This enhanced perinatal wound healing in mice comports with our understanding of fetal wound healing in other systems, which occurs in a scarless fashion with more robust regeneration (Gurtner et al., 2008). Combining the results of these two studies, it appears that very young mice have an enhanced capacity for regeneration that is attenuated shortly thereafter. Yet, the capacity for regeneration in extremely aged animals remains unaddressed by these experiments.

There are reasons to believe WIHN may be less robust in animals at the end of their lifespan. First, wound healing responses in very old mice (∼2 years) differ markedly from their young counterparts, recapitulating the dermal fragility and poor wound healing observed in elderly humans (Keyes et al., 2016). When compared to young mice (2–4 month), elderly mice (22–24 months) showed decreased kinetics of wound closure and decreased capacity for keratinocyte proliferation. Skin thickness in these older mice was also attenuated. As discussed below, major cytokine pathways driving this decline in cutaneous wound healing are also implicated in WIHN. Further, this same group examined the response of young (2 month) and old (2 year) mice to partial thickness cutaneous wounds (Ge et al., 2020). Skin was removed to the depth of epidermis and partial dermis but with hair follicle bulges left intact. Interestingly, while hair follicle stem cells (HFSCs) were found to be present in near normal numbers in the old mice and had retained their identity, they engaged in aberrant cycles of hair follicle renewal in response to wounding. As discussed below, since some of these same HFSCs are involved in hair follicle neogenesis, it will be interesting to examine whether similar abortive regeneration occurs in response to full thickness wounds that normally produce WIHN.

## Cellular Processes in WIHN

Disparate cellular elements coordinate the regeneration of epidermis, dermis, and hair follicles during WIHN. These include keratinocytes and hair follicle stem cells (HFSCs) concentrated in different areas of the interfollicular epidermis and hair follicle (HF). Here we consider what is known about the age-related dynamics of these cells in homeostasis and wound repair. Distinct lineages of HFSCs have a particular niche both during the normal HF cycle and upon wounding. The ability of HFSCs to effectively maintain this niche can be affected by age-related factors such as changing gene expression programs, stem cell location, DNA and oxidative damage, and extrinsic factors in the tissue microenvironment.

### Hair Follicle Stem Cells

Hair follicle stem cells are clustered in specific regions of the hair follicle and surrounding skin including the bulge (Krt15^+^, CD34^+^, Lgr5^+^, Sox9, Tcf3), isthmus (Lgr6^+^, Plet1^+^), infundibulum (Lrig1^+^) interfollicular epidermis (Lgr6^+^), and sebaceous gland (Lgr6^+^, Blimp1^+^, Gata6 lineage) (Dekoninck and Blanpain, 2019; Wier and Garza, 2020). The bulge is the area of the hair follicle that contains long-lived HFSCs responsible for hair renewal throughout life – cycles of anagen (growth), catagen (degeneration), and telogen (resting stage) (Cotsarelis et al., 1990; Blanpain and Fuchs, 2006). Given their key early role in healing of the intrafollicular epidermis, it was surprising that Krt15^+^ bulge stem cells from adjacent HFs were shown to be insignificant contributors to hair follicle regeneration in WIHN (Ito et al., 2005, 2007). As these cells are critical for normal hair follicle cycling, their lack of contribution to WIHN reveals an important difference between physiologic renewal and damage-induced regeneration. It was later shown that certain bulge stem cells were not essential for WIHN as long as there remained a functional interaction between the epithelium and mesenchymal dermal papilla (Ito et al., 2007; Rompolas et al., 2013). The latter study showed that following laser ablation of the bulge, distant epithelial cells above the bulge (infundibulum) contributed to creating the missing bulge compartment and subsequent hair growth (Rompolas et al., 2013), consistent with a role for stem cells of the isthmus in contributing to neogenic follicles (Snippert et al., 2010). This suggests that non-bulge stem cells have greater lineage plasticity following wounding and are primary contributors to WIHN.

Stem cells characterized by other marks in the bulge and isthmus have been shown to be important contributors to WIHN. Leucine-rich-repeat-containing-G-protein-coupled-receptor (Lgr) cells are critical for physiologic renewal of stratified epithelia and for regeneration in WIHN. Lgr6^+^ cells are the most primitive epidermal stem cell lineage, capable of generating many if not all epidermal structures during development and contributing to maintenance of the IFE in homeostatic conditions. Lineage tracing experiments showed that descendants of these cells contribute to newly generated hair follicles and IFE during WIHN (Snippert et al., 2010). Lgr5^+^ SCs are derived from the Lgr6^+^ pool early in the life and become independent soon thereafter. Lgr5^+^ cells contribute to all hair lineages but not to sweat glands or interfollicular epidermis (Snippert et al., 2010). Wang et al. (2017) showed through lineage tracing during WIHN that descendants of Lgr5^+^ epithelial stem cells were present in 40% of the regenerated HFs and that Lgr5^+^ cell depletion reduced WIHN by over 50%.

Recent studies using high throughput lineage tracing of Lgr5^+^ and Lgr6^+^ SCs and transcriptomic analysis capture the molecular adaptation of SC progeny from different niches during wound healing. The results demonstrate remarkable plasticity in HFSCs whereby wounds productive of WIHN displayed downregulation of bulge stem cell gene expression and induction of an IFE-like signature in Lgr5^+^ HF progeny (Joost et al., 2018). This IFE-like signature and improved regenerative ability largely resulted from an increase in surface receptors that could interact with the wound environment. Lgr6^+^ progeny possessed that ability even before wounding (Joost et al., 2018). This increase in regenerative capacity with improved crosstalk between HFSCs and the wound environment underscores the importance of the microenvironment on the plasticity of HFSCs. Interestingly, similar lineage infidelity is also evident in HFSCs mobilized in both young and old animals during the healing response to partial thickness wounds (Ge et al., 2020). In aged animals, these HFSCs migrate from the bulge and express both IFE and HF markers. However, these cells fail to complete hair follicle renewal resulting in smaller, morphologically variant follicles. It is important to note that the partial thickness wounding utilized in these experiments does not completely destroy hair follicles so the failure here is one of hair follicle renewal rather than a failure of *de novo* hair follicle regeneration. Therefore, it will be interesting to see whether the same blurring of IFE and HFSC transcriptional programs is maintained in aging animals during WIHN and whether there are functional consequences for number and morphology of neogenic follicles.

Insights gleaned from non-hair follicle systems are also shedding light on changes in the function of Lgr5^+^ and Lgr6^+^ stem cells with aging. In the hyponychium – the portion of the nail bed responsible for nail renewal – absolute numbers of Lgr6^+^ cells do not seem to vary in young and aged human samples (Shi et al., 2018). However, the younger Lgr6^+^ cells demonstrated a greater proliferative capacity than older cells, and the younger nails had higher growth rates. It is in the intestinal system that Lgr + epithelial stem cells first came to prominence due to their roles in mucosal turnover and intestinal organoid formation. In studies comparing Lgr5^+^ intestinal stem cells in young mice (2 month) and old mice (22–24 month) there was no significant change in the number of intestinal crypts with Lgr5^+^ cells nor in their relative percentage compared to other cell types (Nalapareddy et al., 2017). However, there was a decline in Lgr5^+^ SC function – migratory capacity and ability to contribute to crypt morphology and viability – and turnover in the old mice. Similarly, in both mouse and human samples, the numbers of Lgr5^+^ intestinal stem cells and their proliferative capacity were undiminished with age (Pentinmikko et al., 2019). Yet the regenerative capacity of these cells as judged by organoid formation was markedly decreased. Strikingly, this change was due to the production of a soluble Wnt inhibitor – notum – by adjacent Paneth cells in the crypt. The observations of Lgr5^+^ and Lgr6^+^ SCs in these systems suggest that we can expect greater age-related changes in the response of these cells to regenerative stimuli during WIHN rather than changes in their absolute number.

Changes in HFSC microenvironment can result in significant attenuation of hair follicle cycling with aging. Ge et al. (2020) used scRNAseq to compare the transcriptomic identities of bulge, hair germ, isthmus, infundibular, sebaceous gland, basal epidermal, and supra-basal epidermal stem cells of 2-mo-old (young) and 2-y-old (aged) mice during telogen. There was little noticeable difference between the cellular pattern of young and aged epithelia, suggesting no age-related blurring of cellular states. Specifically, aged bulge HFSCs did not show signs of lineage infidelity marked by induction of epidermal genes or loss of key HF identity transcripts. However, and in agreement with their earlier studies, transcriptomic analysis of young and aged cells showed that aged HFSCs had significant changes in expression profiles of ECM, ECM-remodeling genes, basement membrane, and secreted signaling factors (Keyes et al., 2013; Ge et al., 2020). Strikingly, modulating the stem cell environment by commingling these cells with cells from neonatal dermis allowed even aged HFSCs to regenerate hair follicles when transplanted onto nude mice. This suggests that certain secreted factors or ECM components in the neonatal dermis can rejuvenate aged HFSCs.

### Fibroblasts

The importance of fibroblasts to regeneration of follicles and subdermal tissues has been demonstrated in several studies. Emerging evidence points to a role for fibroblasts in the age-related attenuation of WIHN (Gay et al., 2013; Rognoni et al., 2016). Two primary lineages of fibroblasts include upper dermal (papillary) fibroblasts marked by Lrig1^+^ and responsible for regulation of hair growth and the arrector pili muscle, and lower dermal (reticular) fibroblasts that secrete fibrillar ECM (Driskell et al., 2013). Upon wounding, lower dermal fibroblasts proliferate first, and start producing collagen. After this and only following re-epithelialization, the upper dermal fibroblasts migrate to regenerate the papillary dermis that is important for HF formation and growth (Driskell et al., 2013). Rognoni et al. (2016) found that following 2 mm circular full-thickness wounds on the back of P2 and P21 mice, P21 mice formed far fewer HFs than did P2 mice. Interestingly between P2 and P50, fibroblast density was lower in the dermis, but the rate of proliferation was the same. This was observed even in wound beds that have a lower ability to support HF neogenesis such as in the tail. Interestingly, the post-natal decline in WIHN correlated with a dramatic reduction in Lrig1^+^ papillary fibroblasts around P10 suggesting that the balance between the two lineages of fibroblast is more critical for regeneration than absolute numbers. This increase in recruitment of lower lineage fibroblasts at the expense of papillary fibroblasts occurs in a Wnt-dependent fashion (Rognoni et al., 2016).

While the data from Rognoni et al. (2016) compare regeneration in neonatal and young adult mice, their observations of fibroblast dynamics comport with those seen in older mice. In a study comparing the relative thickness of papillary dermis and reticular dermis of young (2 month), middle-aged (9 month), and old (18 month) mice, the old mice had a decrease in the thickness and density of the papillary dermis, but a significant increase in thickness of the reticular layer (Salzer et al., 2018). These changes were already visible albeit not as pronounced in the middle-aged mice. Transcriptomic analysis of fibroblasts from these animals revealed that CD26 + old papillary fibroblasts differentially expressed genes involved in promoting cytoskeletal extensions, cell contacts, and cell-cell repulsion signals (Salzer et al., 2018). These data support a mechanism in which aged dermal fibroblasts prefer to fill the empty dermal space created by surrounding dying fibroblasts with membrane protrusion rather than through proliferation (Salzer et al., 2018), a conclusion supported by tracking fibroblasts over time with intravital imaging (Marsh et al., 2018). Interestingly, membrane extension rather than proliferation is also utilized by these aged fibroblasts in response to wounding. Taken together, these data suggest that the decreased density of papillary fibroblasts that occurs post-natally continues into old age with likely attendant consequences for diminished WIHN. Augmenting this fibroblast population could lead to more robust regeneration in aging animals.

### Inflammatory Cells

Inflammatory cells including dermal γ-δ T-cells, dendritic epidermal T-cells (DETCs), and macrophages have emerged as important regulators of WIHN. In mice, γ-δ T-cells (named for the two chains that make up their T-cell receptor) are present in substantial numbers in epidermis and play a key role in triggering WIHN (Gay et al., 2013). Specifically, dermal γ-δ T-cells secrete fibroblast growth factor 9 (Fgf9) which induces hair follicle neogenesis after wounding in a Wnt-dependent fashion (Gay et al., 2013). Reducing Fgf9 expression by using mice lacking γ-δ T-cells or mice with Fgf9-deleted T cells led to a dramatic reduction in WIHN with 60% fewer regenerated hair follicles (Gay et al., 2013). By contrast, overexpression of FGF9 resulted in a two- to threefold increase in the number of neogenic hair follicles. In a different study, after making full-thickness dorsal skin wounds in mice, the absolute copy numbers of mRNAs encoding various fibroblast growth factors were quantified in young adult mice (2 month) and old mice (9 month) (Komi-Kuramochi et al., 2005). In young mice, Fgf9 mRNA copy numbers were fivefold higher than in the aged animals. It is interesting to note that the proportion of γ-δ T-cells amongst CD45^+^ T-cells in the wound site did not change after full-thickness wounding in young (2–4 month) versus aged (22–24 month) mice (Keyes et al., 2016). These data raise the intriguing possibility that downregulation of Fgf9 secretion by aging γ-δ T-cells rather than a reduction in cell numbers may attenuate WIHN in aging animals.

## Signaling Pathways

### Wnt/β-Catenin Signaling

Since its characterization by Ito et al. (2007), WIHN has been shown to be driven by a number of signaling pathways including Wnt/β-catenin, Fgf9, Shh, IL-6/STAT3, and retinoic acid pathways. The canonical Wnt/β-catenin pathway was the first and most extensively characterized pathway to be implicated in WIHN (Ito et al., 2007). Given the key role of Wnt signaling in establishing hair follicles during embryonic development, it is not surprising that this pathway is reactivated during neogenic follicle development in adults. Ito originally found that expression of Wnt inhibitors in healing epidermis abrogated WIHN, while upregulation of Wnt/β-catenin signaling in the same system led to an increase in neogenic follicle numbers (Ito et al., 2007). In subsequent work, Wnt/β-catenin activation has emerged as a key node integrating roles of disparate cellular and signaling events to promote follicle neogenesis in response to wounding. Gay et al. (2013) found that dermal γ-δ T-cells secrete Fgf9 which triggers Wnt2a secretion and subsequent activation of myofibroblasts. This results in a positive feedback loop by which activated fibroblasts express more Fgf9 and further upregulate dermal Wnts (Gay et al., 2013). Since the γ-δ T-cells of aged mice secrete considerably less Fgf9 than their young counterparts, the decrease in subsequent dermal Wnt activation may account for a decline in WIHN with aging (Komi-Kuramochi et al., 2005). Surprisingly, given the pro-regenerative role of Wnt/β-catenin signaling in the epidermis, increasing Wnt/β-catenin signaling in dermal fibroblasts of young adult mice led to attenuation of WIHN as compared with neonates (Rognoni et al., 2016). The increased activation of β-catenin in the adult mice resulted in increased recruitment of lower dermal fibroblasts, which are unable to induce HF formation, at the expense of papillary dermal fibroblast as discussed above (Rognoni et al., 2016). Given the complex interplay between epithelia and fibroblasts in formation of the dermal papilla and subsequent events in hair follicle morphogenesis, restriction of Wnt morphogens to particular geographies may be needed to promote regeneration. In addition to these spatial dynamics, temporal dynamics in Wnt/β-catenin signaling within the epidermal compartment also come into play. Castilho et al. (2009) found that prolonged Wnt1 expression induced rapid growth of HFs but subsequently led to the exhaustion of HFSCs as seen through the diminution of CD34^+^ cells in the HFSC niche and activation of cell senescence pathways. Indeed, the silencing of this stem cell population upon prolonged Wnt exposure in older animals that have seen numerous hair cycles seems to serve a protective role by preventing these cells from nucleating follicular cancers. Therefore, far from being an on/off switch for promoting regeneration, Wnt/β-catenin signaling must be carefully governed in time and space to generate optimal WIHN.

### Hedgehog Signaling

Sonic hedgehog (Shh) is a critical signaling molecule in the hair cycle and in WIHN. Knocking out Musashi 2 (Msi2), an evolutionarily conserved RNA binding protein that represses Shh signaling, in tissue-specific stem cells resulted in a dramatic reduction of *de novo* hair follicle regeneration (Sugiyama-Nakagiri et al., 2006; Ma et al., 2017). Lim et al. (2018) showed that Shh signaling is essential for WIHN and that Shh activation shifts dermal fibroblast fate toward the dermal papilla. This suggests that reticular dermal fibroblasts that promote scarring can be redirected to a regenerative phenotype, similar to the fate switch that embryonic fibroblasts must undergo when encountering epidermal hair follicle placodes to generate hair follicles in development. Further underscoring the importance of hedgehog (Hh) signaling in adult hair follicle regeneration, Sun et al. (2020) demonstrated that exogenous activation of the pathway in cutaneous epithelia and stroma can lead to *de novo* hair follicle formation. This was observed even in normally non-hair-bearing areas of the mouse such as the paw and occurred in the absence of wounding. While the Hh pathway is critical for follicular morphogenesis in embryonic development and adult regeneration, there have been no studies comparing Shh expression in young and old mice during WIHN. However, age-related decrease Shh expression has been observed in other systems including endometrium where decreased Shh levels lead to impaired endometrial stem cell activity and poor endometrial renewal (Cho et al., 2019). Interestingly, in HFSCs isolated from hair follicle bulges in young and old human scalp biopsies, Hh pathway genes were expressed at similar levels (Rittie et al., 2009). Whether this similarity in gene expression occurs during normal homeostasis only or whether it also obtains in the context of wounding remains to be seen. Further, given the interplay between Shh and other signaling systems in skin that demonstrate age-related changes such as Wnts, deconvoluting the role of Shh in regeneration and aging will be an important area for future investigation (Ouspenskaia et al., 2016).

### dsRNA/IL-6/STAT3/RA

The innate immune receptor Toll-Like Receptor 3 (TLR3) is an important driver of WIHN (Nelson et al., 2015). Activation of TLR3 signaling in keratinocytes promotes expression of stem cell markers and leads to induction of key hair follicle morphogenic programs. TLR3 responds to dsRNA species which are released by damaged or dying cells, thereby serving as a direct link between damage sensing and regeneration. Downstream effectors of TLR3 in WIHN include interleukin 6 (IL-6) and signal transducer and activator 3 (STAT3) (Nelson et al., 2015). Nelson et al. found that HF generation after full-thickness wounding in TLR3 null mice was dramatically reduced compared to controls. Comparing gene expression signatures from healed wound beds of mice with robust HF regeneration to those of mice with poor regeneration, they found that IL-6 was significantly upregulated in the prolific regenerators. They further showed that this IL-6 induction was TLR3 dependent with TLR3-null mice having far less IL-6 mRNA post-wounding than strain-matched controls. Activation of the IL-6 receptor results in the phosphorylation of STAT3 (pSTAT3), which leads to its nuclear translocation and transcriptional activation (Heinrich et al., 2003). When mice were treated with cucurbitacin I, a highly selective pharmacological inhibitor of STAT3, they had a more than threefold decrease in the number of regenerated HFs following wounding showing that STAT3 is functionally required for WIHN (Nelson et al., 2015). In summary, TLR activation in response to wounding leads to IL-6 production and STAT3 phosphorylation, activating a STAT3-dependent gene expression program to promote WIHN. This conclusion agrees with recent studies that have characterized other IL-6 type molecules such as IL-36α that activate STAT3 and induce WIHN (Gong et al., 2020).

**FIGURE 1:**
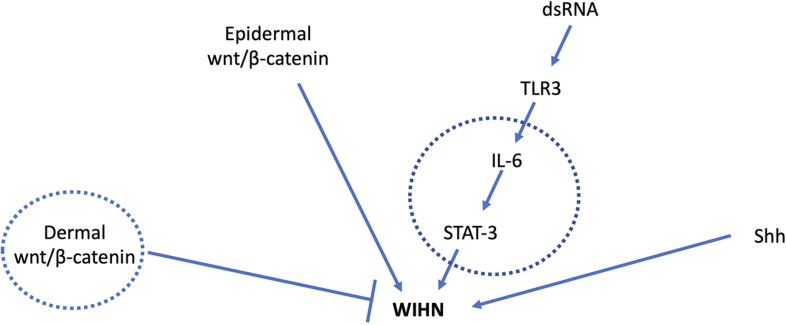
Major signal transduction pathways involved in WIHN. Circles indicate mediators known to have age-related changes. Young adult mice with high wnt/β-catenin levels in the dermis had fewer pro-regenerative fibroblasts and impaired WIHN (Rognoni et al., 2016). IL-6 levels are lower in aged mouse skin with impaired wound healing (Keyes et al., 2016), and low IL-6/STAT-3 is also observed in mice with low WIHN (Nelson et al., 2015). Shh signaling is a major driver of developmental and regenerative hair follicle morphogenesis. Age-related changes in Shh during WIHN have not been demonstrated.

Strikingly, the same IL-6/STAT3 pathway that governs WIHN is implicated in impaired cutaneous wound healing in aged mice. In comparison to young mice (2–4 month), aged mice (22–24 month) had significantly slowed wound healing and diminished capacity for keratinocyte proliferation and migration. This correlated with a 4.5-fold reduction in IL-6 transcription in keratinocytes of aged mice (Keyes et al., 2016). Exogenous application of IL-6 rescued the poor wound healing kinetics in aged mice, reminiscent of its ability to augment WIHN (Nelson et al. and Figure 1). Additionally, the age-related attenuation in wound healing observed by Keyes et al. (2016) is mediated largely through STAT3 signaling in dendritic epithelial T cells (DETCs), a major resident subtype of cutaneous γ-δ T-cells. Keyes et al. (2016) found that in the unwounded state, DETC numbers were unaffected by STAT3 loss. However, after wounding, DETC number declined dramatically in the skin of STAT3 cKO mice. Thus, the epithelial-DTEC crosstalk examined here is critical for normal wound healing, converges on IL-6/STAT3 signaling, and is attenuated in aged animals with impaired wound healing. While this study did not directly assess hair follicle regeneration in young and old mice, it is interesting to speculate that this too will be reduced given its common dependence on IL-6 and STAT3.

Retinoic acid (RA), was recently discovered to induce WIHN following TLR3 activation by dsRNA (Kim et al., 2019). Kim et al. (2019) found that TLR3 signaling induces RA accumulation, and that exogenous RA can rescue deficient hair follicle regeneration in TLR3 deleted mice. It is interesting to note that the TLR3/RA pathway is connected to the STAT3 pathway. STAT3 stimulates aldehyde dehydrogenase1a3 which converts retinaldehyde to RA (Canino et al., 2015). Furthermore, laser treatment of skin, which can be done for facial rejuvenation, results in elevation of RA synthesis (Kim et al., 2019). This suggests that the rejuvenation of aged human skin likely works by a similar mechanism involving dsRNA/damage-induced RA synthesis. RA-based compounds known as retinoids have also been used clinically for decades to rejuvenate aging skin and function by both increasing collagen production by stimulating fibroblasts and inhibiting ECM degradation by matrix metallopeptidases (MMPs) (Lateef et al., 2004; Mukherjee et al., 2006). Aged skin has been shown to have an upregulation of MMPs as well as decreased collagen production due to the age-dependent alteration of fibroblast function (Varani et al., 2006). Thus, an interesting avenue of investigation would be to see if the TLR3/RA pathway is downregulated or compromised during WIHN in aged mice.

### Hippo Signaling

The ECM may modulate WIHN via mechanotransduction activation of the transcriptional cofactor Yes-associate protein (YAP). YAP is a key effector of the Hippo signaling pathway that is involved in organ size control (Pan, 2010). Physico-mechanical stresses applied to ECM can result in changes in substrate stiffness. These forces can in turn alter cytoskeletal dynamics on attached cells leading to activation and nuclear translocation of YAP (Dupont et al., 2011). In uninjured skin, YAP is activated in HFs and epidermal cells but not in surrounding dermal cells (Lee et al., 2014). Upon wounding, YAP localized to the nucleus in dermal skin cells in 2 days with expression throughout the wound area by 7 days (Lee et al., 2014). This is likely due not only to the inhibition of the Hippo pathway due to decreased cell-crowding but also to mechanical cues that act independently from the Hippo pathway (Dupont et al., 2011). Wound closure has been shown to be delayed following downregulation of YAP by siRNA treatment and is likely a result of decreased activity and production of TGF-ß1, a downstream factor activated by YAP (Lee et al., 2014). During HF development, transgenic activation of YAP leads to increased proliferation of basal epidermal SCs at the expense of terminal differentiation (Zhang et al., 2011). Furthermore, the study also found that nuclear YAP progressively declines with age and correlates with the proliferative potential of epidermal SCs. Given the known role of these cells in regeneration of HFs and IFE in WIHN, it will be interesting to see if this age-related dysregulation of YAP activation leads to impaired WIHN in older animals.

## Remaining Questions and Conclusion

Impaired capacity for renewal and regeneration – seen notably in cutaneous wound healing (Keyes et al., 2016) – is an important source of age-related morbidity. As one of the few truly regenerative phenomena in adult mammals, WIHN represents a powerful lens with which to investigate this decline and offer solutions for its reversal. We have described a number of cellular – HFSC and epidermal stem cell, fibroblast, T cell – and molecular – Wnt, IL-6/STAT3, Hippo pathway – players in WIHN that show alterations with age (Figure 1). The production of neogenic follicles itself declines from the neonatal period through youth in mice, paralleling a later decline in the capacity for wound-induced hair follicle renewal (Ge et al., 2020) and for cutaneous wound healing (Keyes et al., 2016). This raises the tantalizing possibility that WIHN may continue to decline throughout age, though no rigorous studies have been performed to test this. Quantitative investigation of WIHN in middle aged and extremely aged animals may better elucidate regenerative decline and define its mechanisms.

Such mechanisms fall into three broad categories. First, older animals may be less sensitive to damage-induced molecular cues that initiate the regeneration cascade. Second, such animals may fail to mobilize precursor cells to rebuild missing structures. Given the number of cells that seem to contribute to regenerated hair follicles – keratinocytes, epidermal stem cells, and HFSCs that constitute the new follicles, and immune cells that coordinate their response – there are many places where regeneration could break down. Finally, precursors even when appropriately mobilized may fail to reconstitute *de novo* follicles resulting in frustrated, incomplete morphogenesis. Indeed, the failure of otherwise present and genetically stable HFSCs to create new hair follicles is evident in aged mice subjected to partial thickness wounding, suggesting that a similar situation may obtain in WIHN (Ge et al., 2020). Discriminating between these three possibilities or combinations of them will provide a rich ground for future studies and may afford insights into improving regenerative capacity with age.

## Author Contributions

SR defined the structure of the manuscript. MB and SR wrote the manuscript. SR and LG edited the manuscript. All authors contributed to the article and approved the submitted version.

## Conflict of Interest

The authors declare that the research was conducted in the absence of any commercial or financial relationships that could be construed as a potential conflict of interest.
